# Nasal Potential Difference in Cystic Fibrosis considering Severe *CFTR* Mutations

**DOI:** 10.1155/2015/306825

**Published:** 2015-01-15

**Authors:** Ronny Tah Yen Ng, Fernando Augusto de Lima Marson, Jose Dirceu Ribeiro, Antonio Fernando Ribeiro, Carmen Silvia Bertuzzo, Maria Angela Gonçalves de Oliveira Ribeiro, Silvana Dalge Severino, Eulalia Sakano

**Affiliations:** ^1^Department of Otolaryngology, State University of Campinas (Unicamp), 13083-887 Campinas, SP, Brazil; ^2^Department of Pediatrics, Center for Pediatrics Research (CIPED), State University of Campinas (Unicamp), 13083-887 Campinas, SP, Brazil; ^3^Department of Medical Genetics, State University of Campinas (Unicamp), 13083-887 Campinas, SP, Brazil

## Abstract

The gold standard for diagnosing cystic fibrosis (CF) is a sweat chloride value above 60 mEq/L. However, this historical and important tool has limitations; other techniques should be studied, including the nasal potential difference (NPD) test.* CFTR* gene sequencing can identify* CFTR* mutations, but this method is time-consuming and too expensive to be used in all CF centers. The present study compared CF patients with two classes I-III* CFTR* mutations (10 patients) (G1), CF patients with classes IV-VI* CFTR* mutations (five patients) (G2), and 21 healthy subjects (G3). The CF patients and healthy subjects also underwent the NPD test. A statistical analysis was performed using the Mann-Whitney, Kruskal-Wallis, *χ* ^2^, and Fisher's exact tests, *α* = 0.05. No differences were observed between the CF patients and healthy controls for the PDMax, Δamiloride, and Δchloride + free + amiloride markers from the NPD test. For the finger value, a difference between G2 and G3 was described. The Wilschanski index values were different between G1 and G3. In conclusion, our data showed that NPD is useful for CF diagnosis when classes I-III* CFTR* mutations are screened. However, if classes IV-VI are considered, the NPD test showed an overlap in values with healthy subjects.

## 1. Introduction

Cystic fibrosis (CF) (MIM: number 219700) is an autosomal disorder with high clinical variability that is associated with* CFTR* mutations, environmental effects, and modifier genes [1–12]. Since 1989 [13–15], the discovery of the* CFTR* (cystic fibrosis transmembrane regulator; 7q31.2 region) gene and medical advances in CF knowledge have shown that CF is a disease with complex clinical presentation [1–3]. Since 1959, CF diagnosis has been obtained using the Gibson and Cooke test, that is, the sweat test [16]. The sweat test is an important tool that provides a CF diagnosis in the majority of patients at a low cost. However, in cases of nonclassic CF disease, specifically cases caused by class IV, V, or VI* CFTR* mutations [17–19], patients can show normal chloride values in their sweat [20].

Complete* CFTR* gene sequencing can provide a CF diagnosis. However, it is expensive, is time-consuming, and may not be possible in all CF centers around the world. Therefore, other tools are being studied for CF diagnosis, including the following: (i) the concentrations of chloride and sodium in the saliva [21]; (ii) *β*-adrenergic sweat secretion [22]; (iii) measurements of CFTR-mediated chlorite (Cl) secretion in human rectal biopsies [20]; (iv) newborn screening (NBS) by assessing immunoreactive trypsinogen (IRT) (that is, following a positive IRT, the sweat test should be performed for CF diagnosis confirmation [23]); and (v) sequencing of the entire* CFTR* gene [24]. In developing countries, a CF diagnosis can be obtained by measuring chloride and sodium levels and usually by performing an F508del (cDNA: c.1521_1523delCTT) mutation screening [25, 26].

The nasal potential difference (NPD) measurement is a diagnostic method that is sensitive and specific, validates CFTR function, and provides in vivo evidence of abnormal ion transport due to the dysfunction of the CFTR protein in nasal epithelial cells. For clinical management, a comparison among several CFTR biomarkers shows that NPD reflects the CFTR function in the respiratory tract, an organ strongly related to CF survival. However, NPD has not been extensively assessed for its reproducibility and reliability for diagnosis. There is an absence of validation for diagnosis, and in the literature there is some correlation with respiratory clinical endpoints. However, this tool is by far the most extensively validated biomarker [27] and was used successfully to measure CFTR modulator therapy with ivacaftor in patients with G551D* CFTR* mutations [28, 29]. NPD is used to measure the voltage across the nasal epithelium which results from transepithelial ion transport and partially reflects CFTR function [30]. Electrophysiological abnormalities in CF were described nearly 50 years ago and correlate with features of the CF phenotype.

Considering CF diagnoses in an admixed population, our study compared three groups of subjects (patients with two class I, II, or III* CFTR* mutations (group A); patients with at least one class IV, V, or VI* CFTR* mutation (group B); healthy subjects (group C)) in association with NPD. The aim of the study was to verify the effectiveness of NPD to differentiate healthy individuals from those with severe CF mutations and with mild CF mutations.

## 2. Materials and Methods

### 2.1. Cystic Fibrosis Patients and Control Subjects

The CF patient group initially included 21 patients based on CF clinical characteristics and sodium and chloride values above 60 mEq/L on the sweat test. F508del screening and* CFTR* sequencing were performed in 15 of the 21 CF patients; six (28.57%) patients were excluded. The CF population ultimately included 15 CF patients (group A: 10 patients and group B: five patients). The* CFTR* genotype was used to assign patients to the groups and to enable a comparison between classic and nonclassic CF. We also enrolled 21 healthy subjects without CF or other diseases and with no familial history of CF (group C).

Individual factors such as smoking, previous sinus surgery, nasal polyposis, or acute upper respiratory tract infection have a negative influence on CFTR response [27]. In this context, all patients and healthy controls with these conditions were excluded from our analyses. The project was approved by the University Ethics Committee (number 279/2007), and all of the patients and/or their guardians signed an informed consent before inclusion in the study.

### 2.2. Molecular Analysis

A peripheral blood sample was collected from each subject. Genomic DNA was obtained by direct extraction from peripheral blood lymphocytes according to standard procedures [31].* CFTR* mutations were determined in the following order: F508del identification using the primers forward 5′-GGC ACC ATT AAA GAA AAT ATC-3′ and reverse 5′-TGG CAT GCT TTG ATG ACG C-3′ [25, 26];* CFTR* exon sequencing, including exon/intron boundaries, performed as previously described [24, 32, 33]; duplication, deletion, and LOH identification using the SALSA MLPA Kit P091-C1 CFTR-MRC-Holland (MRC-Holland, Willem Schoutenstraat, DL Amsterdam, Netherlands) performed according to the manufacturer instructions; and 1584–18672 pb A>G (intron 10) identification performed as previously described [34].

### 2.3. Nasal Potential Difference Measurements

For the NPD measurement, we used a high-impedance voltmeter with a storage capacity for 100 data points (Knick Portamess 913, E) and AgCl electrodes with low impedance (less than 10^3^ ohms). The voltmeter was set to record readings every 10 sec, and the measurements were transferred to a computer and stored using Paraly SW105 software (ProMinent Dosiertechnik GmbH, Germany).

Electrode 1 (reference) was positioned and fixed on the anterior left forearm in scarified skin by a diamond-tip drill for odontology use. Electrode 2 (exploring electrode) was positioned within of the larger channel of a silicone probe filled with a cream conductor (Sigma Electrode Cream, Parker Laboratories).

We conducted finger measurements with electrode 2 between the thumb and index finger, which were soaked in the conductor cream, and we then positioned the probe tip 3.0 cm, 2.0 cm, 1.5 cm, 1.0 cm, and 0.5 cm below and posterior to the right inferior turbinate with the aid of a rigid nasal endoscope. NPD measurements in all positions were achieved when the measurement had stabilized for more than five sec. The maximum potential difference (PDMax) was considered the most negative measure. The probe was released at a position of 1.5 cm.

The reference electrode was an isotonic NaCl- or Ringer's saline-perfused bridge in the subcutaneous space of the lower arm. The reference bridge was 19 to 24 ga. needle containing isotonic Ringer's saline. The exploring electrode was an isotonic NaCl-/Ringer's saline-perfused exploring bridge positioned on the airway surface. Exploring bridges were prepared either by filling lengths of polyethylene (PE) tubing (PE-50 to PE-160) with 3 M KCl in 4% agar or by a fluid-filled double-lumen catheter continuously perfused with warmed (24–37°C) gassed isotonic NaCl/Ringer's saline (0.2–0.4 mL/min). Contact with the nasal surface was ensured by perfusion. The exploring bridge consisted of a vinyl catheter (e.g., an umbilical vessel catheter, 5Ch 1.7 mm). Most catheters can be used repeatedly following gas sterilization [35].

For the probe, five solutions were infused inside the nasal cavity with a flow rate of five mL/min, which was controlled by an infusion pump previously heated to a final temperature of 37°C. Each solution was sequentially infused for three min with no pauses between the infusions. Solution A was custom Ringer's: 135 mM NaCl; 1.2 mM MgCl_2_; 2.25 mM CaCl_2_; 2.4 mM K_2_HPO_4_; and 0.4 mM KH_2_PO_4_. Solution B was 0.1 mM amiloride hydrochloride (HCl) in Ringer's solution. Solution C was zero Cl^−^ solution (+ amiloride): 135 mM sodium gluconate, 1.2 mM MgSO_4_, 2.2 mM calcium gluconate, 2.4 mM K_2_HPO_4_, 0.4 mM KH_2_PO_4_, and 0.1 mM amiloride HCl. Solution D was 0.01 mM isoproterenol HCl in solution C (caution: vials of isoproterenol contain Cl^−^). Solution E was 0.1 mM ATP in solution D. The mean pH was 7.4, with a range of 7.0–7.6. Solutions A, B, and C may have been refrigerated for up to three months or frozen for up to six months, whereas solutions D and E were freshly prepared within two h prior to use. Prior to use, all the solutions were filtered with 0.22 *μ*m filter [35].

The voltmeter was programmed to obtain an NPD measurement every 10 seconds, for a total of 18 measurements for each solution. These values were immediately transferred after each examination to a desktop computer and stored. The NPD test was performed in the right nasal cavity for all the subjects included in the study. The same professional was responsible for performing all the tests. The NPD tests analyzed the following variables: finger, PDMax, Δchloride + free + amiloride, and Wilschanski index. In this context, NPD changes were recorded after perfusion with the following solutions: 100 *μ*M amiloride in saline solution (Δamiloride), a chloride-free solution with 100 *μ*M amiloride (Δchloride-free), and 100 *μ*M amiloride plus 10 *μ*M isoproterenol in a chloride-free solution (Δisoproterenol). The sum of the Δchloride-free and Δisoproterenol values (Δchloride-free-isoproterenol) served as an index of transepithelial CFTR-dependent chloride transport because it reflected the cAMP activation of nasal mucosa chloride permeability. The Wilschanski index was calculated by the following formula: e^Δchloride/Δamiloride^ [36].

The published SOP-NPD (standard operation patronization) [37] was not considered in the present study because this technique was not viable in our center. All test conditions followed previously published NPD requirements [35].

### 2.4. Clinical Markers

We clinically evaluated the CF patients according to the following clinical severity markers: clinical scores (Shwachman-Kulczycki, Kanga, and Bhalla) [38]; body mass index (BMI) (for patients older than 19 years, the BMI = weight/(height)^2^ formula was used; for the remaining patients, the WHO AnthroPlus was used (children from 7 to 19 years old)); patient age and age at diagnosis (according to sodium and chloride alterations in perspiration; first clinical symptoms (digestive and pulmonary disease); the period up to the 1st colonization by* Pseudomonas aeruginosa*; the presence of microorganisms in the sputum (mucoid and nonmucoid* P. aeruginosa*,* Achromobacter xylosoxidans*,* Burkholderia cepacia*, and* Staphylococcus aureus*)); transcutaneous oxygen saturation; pulmonary function tests; CF comorbidities (nasal polyps, osteoporosis, meconium ileus, diabetes mellitus, and pancreatic insufficiency); race; and gender.

The spirometry proof was performed using a model CPFS/D speedometer (Med Graphics, Saint Paul, Minnesota, USA). The data were recorded using BREEZE PF version 3.8 B software for Windows 95/98/NT with the inclusion of the following markers: forced vital capacity (FVC) (%), the forced expiratory volume in the first second (FEV_1_) (%), the ratio of FEV_1_ to FVC, and the forced expiratory flow between 25 and 75% of the FVC (FEF_25–75_%).

### 2.5. Statistical Analysis

The statistical analysis was performed with statistical package for the social sciences (SPSS) software v.21.0 (version 21, SPSS Inc., Chicago, IL). The sample power was determined using GPower 3.0.1 software [39]. Based on the results of the Kruskal-Wallis test as a parameter for population power estimation, for a sample size of 36 subjects, with the *α* error equal to 0.05, and an effect size of 0.5, the statistical power of all the tests performed was 0.812.

The data were compared using the Mann-Whitney (comparison between GA versus GB, GA versus GC, and GB versus GC) and Kruskal-Wallis tests (comparison among GA, GB, and GC) for the NPD variables. To avoid spurious data due to the multiple tests performed [40], the significance level *α* was adjusted by the Bonferroni correction (*α* corrected = 0.05/number of tests).

The clinical markers are shown as percentages for the categorical data and as the means, standard deviations, medians, and minimum and maximum values for the numerical data. The statistical association between the GA and GB was determined using the *χ* ^2^ test and Fisher's exact test for the categorical data and the Mann-Whitney *U* test for the numerical data.

## 3. Results

### 3.1. Patient Characterization and* CFTR* Mutations

Our study included 15 patients (seven (46.67%) males) with CF and 21 healthy controls (seven (33.33%) males). The complete patient characteristics and the comparison between GA and GB are presented in Tables 1 and 2, respectively.

The CF patients were divided into two groups according to mutations identified in the* CFTR* gene. The patients with two class I, II, or III* CFTR* mutations were designated for* group A* (10 CF patients) and those with at least one class IV, V, or VI* CFTR* mutation were designated for* group B* (five CF patients). The healthy subjects were classified as* group C*.

**Figure 1:**
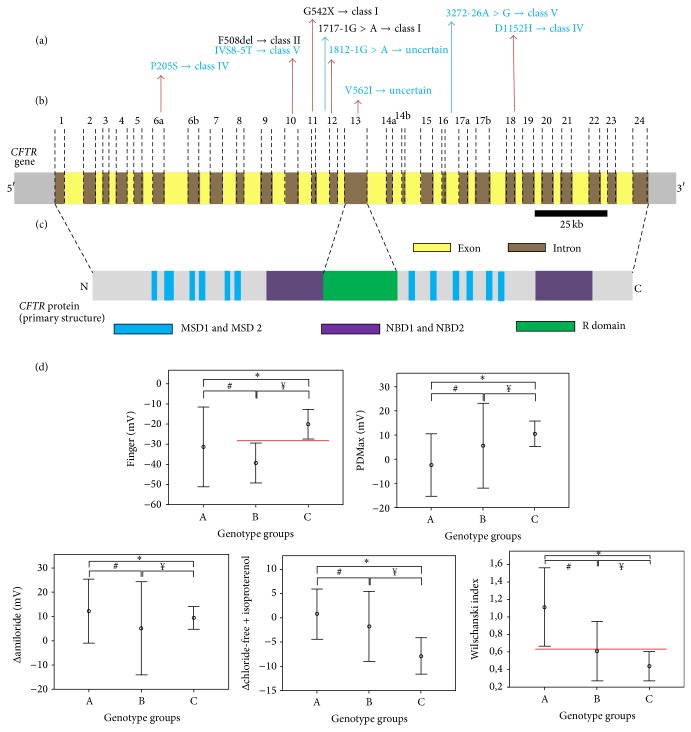
(a)* CFTR* mutations distributed by exon/intron localization and class. (b)* CFTR* gene with exon and intron descriptions. (c) CFTR protein domains. (d) For all the data, consider the following: (A) cystic fibrosis patients with two class I, II, or III* CFTR* mutations; (B) cystic fibrosis patients with two class IV, V, or VI* CFTR* mutations; and (C) healthy subjects. The comparison was made using the Mann-Whitney *U* test considering the following associations: A versus B, A versus C, and B versus C. *α* considered was 0.05. For the finger (mV), ^∗^ *P* = 1; ^#^ *P* =* 0.021*; ^¥^ *P* =* 0.003*. For the PDMax (mV), *P* = 0.426; ^#^ *P* **=** *0.040*; ^¥^ *P* = 0.416. For the Δamiloride (mV), ^∗^ *P* = 0.394; ^#^ *P* = 0.554; ^¥^ *P* = 0.474. For the Δchloride-free + isoproterenol, ^∗^ *P* = 0.390; ^#^ *P* **=** *0.007*; ^¥^ *P* = 0.125. For the Wilschanski index, ^∗^ *P* =* 0.050*; ^#^ *P* =* 0.002*; ^¥^ *P* = 0.345. The maximum difference was observed by finger (B versus C) and for the Wilschanski index (A versus C). CFTR = cystic fibrosis transmembrane regulator, MSD = membrane-spanning domains, NBD = nucleotide-binding domains, R = regulator, and mV = millivolts.

In the GA, the following* CFTR* genotypes were observed: F508del/F508del (eight (80%) patients (class II)), F508del/1717-1G>A (one (10%) patient (mutation class II/class I)), and F508del/G542X (one (10%) patient (mutation class II/class I)) (Figure 1(a)).

In the GB, the following* CFTR* genotypes were observed: F508del/1812-1G>A (one patient (class II mutation/uncertain class)), F508del/3272-26A>G (one patient (mutation class II/class V)), F508del/D1152H (one patient (mutation class II/IV)), F508del/P205S (one patient (mutation class II/IV)), and V562I/IVS8-5T (one patient (uncertain/class V)). Each genotype corresponded to 20% of patients with class IV, V, or VI* CFTR* mutations (Figure 1(a)).

The mutation characteristics are shown in Table 3.

### 3.2. Nasal Potential Difference

The data collected using the NPD test are shown in Table 4 and Figure 1.

Table 4 shows the values of mean, standard deviation, maximum, and minimum as well as confidence intervals of probability from the comparison of the CF patient groups and the healthy subjects for the following NPD variables: finger (*P* = 0.020), PDMax (*P* = 0.111), Δamiloride (*P* = 1), Δchloride-free-isoproterenol (*P* = 0.08), and Wilschanski index (*P* = 0.025).

The complete data are shown in Figure 1(d). For the finger values, there were significant differences between GA versus GB (*P* = 0.021) and GB versus GC (*P* = 0.003). For the PDMax, there was a significant difference in GA versus GB (*P* = 0.04). For Δamiloride, no difference was observed. For Δchloride-free-isoproterenol, there was a significant difference in GA versus GB (*P* = 0.007). For the Wilschanski index, there were significant differences between GA versus GC (*P* = 0.050) and GA versus GB (*P* = 0.002).

## 4. Discussion

Identifying, standardizing, and unifying diagnostic tools for chronic diseases, especially FC, are a constant and ongoing effort in biological research. Even today, diagnosing CF in some individuals remains difficult because CF has numerous phenotypes and genotypes. Therefore, the use of a single tool for diagnosis is complex and dubious. This fact is even more significant in developing countries.

The diagnosis and management of CF in Brazil show variability among CF reference centers. Many problems still exist, such as (i) a high proportion of undiagnosed cases, (ii) delayed diagnosis in many states, (iii) limited services in relation to the actual demand, (iv) a small number of health professionals involved, (iv) disease underestimates by health authorities (low investments and reduced current expenditures), (v) a lack of NBS in most states, and (vi) scientific production that remains limited [41].

To the best of our knowledge, this is the first study conducted in Brazil to evaluate NPD in CF patients diagnosed by screening two* CFTR* mutations in comparison with healthy subjects. Our study showed that measuring NPD can differentiate CF patients with two severe mutations from healthy subjects. However, it was not able to identify differences between patients with class I, II, or III (greater severity) and those with class IV, V, or VI (minor severity)* CFTR* mutations.

Studies have shown that the electronegativity of organs and systems in CF patients is compromised, depending on the* CFTR* mutation class. This causes variations in sweat chloride values among different* CFTR* mutation classes [20]. Measurements of functional CFTR protein in human models for diagnosis, prognosis, and personalized therapy have been initiated and used and are a step forward in the management of CF [27]. Among these tools, the CFTR biomarker-like nasal transepithelial potential (NTP); sweat test; rectal transepithelial Cl^−^ Secretion; and evaporimetry have been studied and stimulated in several centers [27, 42], including our university. These tools have proven useful in the analysis of functional alterations in the CFTR protein in CF patients with class I, II, or III mutations in the* CFTR* gene (severe and classical CF) compared with healthy individuals. However, these tools are not able to separate healthy individuals from patients with genotypes arising from class IV, V, or VI mutations (minor severity and nonclassical CF).

All of these tools show reproducibility/reliability, responsiveness, limitations, feasibility, and availability that limit their application as routine indications in the diagnosis of CF management. Respiratory NPD is by far the most extensively validated* CFTR* biomarker [27]. In contrast, we showed that NTP was useful for differentiating individuals with severe CF from healthy subjects; however, it was not useful for differentiating individuals with mild mutations. Therefore, with the advent of correctors and potentiators for the CFTR protein that are specific for each* CFTR* genotype, this tool became necessary to evaluate the efficiency and effectiveness of new drugs for CF, as has been shown by recent studies. However, its use in clinical practice for CF diagnosis is unclear and should be revised and studied further.

Considering the difficulty of characterizing CF patient groups with class IV, V, or VI mutations, taking into account the possibility of normal TNM values and sweat test, in association with the residual expression of the CFTR protein, new diagnostic tools should be provided, and a potential tool is NPD [28]. If we consider this hypothesis, electrophysiology studies would be important markers to confirm CFTR protein dysfunction but not a diagnostic marker for the disease.

With the introduction of NBS in our state in 2010, children with severe mutations are likely to have early diagnosis confirmed and will be attended at reference centers.

Based on a positive NBS, followed by two chlorine values greater than 60 mEq/L, we obtained CF diagnoses for most CF patients with severe mutations (class I, II, or II). The molecular analysis of* CFTR* mutations may help in increasing specific knowledge about our population, where 61.9% of patients have at least one F508del mutation and 26.7% have two F508del alleles [26].

In our study, the Wilschanski index showed that NPD was significantly different between GA and GC; in this case, it can be useful for CF diagnosis in patients with two class I, II, or III mutations. Therefore, it cannot be inferred that NPD is a diagnostic test for CF, taking into account that patients with class IV, V, or VI mutations (GB) are not differentiated by this technique in comparison with the other groups (GA and GC). The only possible test for certain CF diagnosis in all cases would be* CFTR* gene sequencing.

Considering future prospects, other methods to assess CFTR function have been proposed in the literature, such as beta-adrenergic function studies of the sweat glands [19]. Studies in this line of research are focusing on cases where CF diagnosis is performed and sweat chloride values are normal and it is not possible to identify* CFTR* mutations by sequencing, considering the technical costs.

The limitations of the present study were that (i) NPD was performed without considering the SOP-NPD, (ii) there were a low number of patients in the GB, with negative results for the association that indicates nonerroneous data, and (iii) there was no association with other CF diagnosis tools.

## 5. Conclusions

NPD showed significantly different values between CF patients with two severe* CFTR* mutations of known classes and healthy individuals. However, NPD does not differentiate between those with severe* CFTR* mutations from other CF patients with minor but serious mutations. The NPD should not be used as diagnosis tool for CF patients with class IV-VI* CFTR* mutations. Thus, our proposal that neonatal screening by IRT, followed by the sweat test and screening of the classic F5008del mutation, appears to be satisfactory for the diagnosis of CF in our country. It is quite likely that NPD will be able to assess the improved function of ionic permeability in the cells of the respiratory tract by the action of potentiator CFTR drugs, such as ivacaftor, but not the diagnosis of CF, as our results have shown.

## Figures and Tables

**Table 1:** Clinical and laboratory characteristics of the cystic fibrosis patients enrolled in the study^∗^.

Gender (male)	15	7 (46.7%)
Race (Caucasian)	15	15 (100%)
Age (months)	15	213.13 ± 122.03; 171 (87–443)
Onset of symptoms (months)	14	6.93 ± 13.28; 1 (0–39)
Onset of pulmonary symptoms (months)	14	10.86 ± 19.21; 3 (0–69)
Onset of digestive symptoms (months)	12	19.92 ± 38.57; 1 (0–120)
Diagnosis (months)	14	54.14 ± 101.95; 8,50 (1–378)
Body mass index (normal values)	15	12 (80%)
Nasal polyposis (presence)	15	3 (20%)
Diabetes mellitus (presence)	15	2 (13.3%)
Osteoporosis (presence)	15	3 (20%)
Pancreatic insufficiency (presence)	15	13 (86.7%)
Meconium ileus (presence)	15	3 (20%)
*Pseudomonas aeruginosa *	15	10 (66.7%)
Mucoid *P. aeruginosa *	15	8 (53.3%)
*Achromobacter xylosoxidans *	15	2 (13.3%)
*Burkholderia cepacia *	15	4 (26.7%)
*Staphylococcus aureus *	15	12 (80%)
Weight (kg)	15	43.67 ± 17.02; 34 (21–70)
Height (m)	15	1.58 ± 0.50; 1 (1-2)
Body mass index	15	18.35 ± 2.67; 17.75 (14.33–21.60)
SpO_2_	14	96.07 ± 1.64; 96 (94–98)
Bhalla	11	8.82 ± 4.75; 10 (0–17)
Kanga	13	22.54 ± 12.16; 21 (12–60)
Shwachman-Kulczycki	13	69.23 ± 12.39; 65 (50–90)
FVC	15	84.60 ± 22.02; 82 (57–131)
FEV_1_	15	76.40 ± 25.84; 72 (30–132)
FEV_1_/FVC	14	78.36 ± 19.17; 85 (37–100)
FEF_25–75_%	14	59.50 ± 34.25; 57,50 (70–118)

^∗^The data are shown as *N* (percentage) for the categorical data and as the mean ± standard deviation and the median (minimum and maximum) values for the numerical data. *N*: number of patients; SpO_2_: blood oxygen saturation; FVC: forced vital capacity; FEV_1_: forced expiratory volume in the first second; FEF_25–75_%: forced expiratory flow between 25 and 75% of the FVC.

**Table 2:** Clinical and laboratory characteristics of the cystic fibrosis patients enrolled in the study.

Clinical markers^*^	G1	G2	*P* value
Gender (male)	5	2	1
Race (Caucasian)	10	5	—
Age (months)	210.60 ± 132.01; 146 (87–443)	336.40 ± 119.13; 336 (170–480)	0.099
Onset of symptoms (months)	8.20 ± 15.50; 1 (0–39)	3.75 ± 4.86; 1.50 (1–11)	0.454
Onset of pulmonary symptoms (months)	13.60 ± 22.26; 4 (0–69)	4 ± 4.97; 2.50 (0–11)	0.539
Onset of digestive symptoms (months)	23.70 ± 41.51; 1 (0–120)	1 ± 1.41; 1 (0–2)	0.758
Diagnosis (months)	36.10 ± 47.24; 17 (1–144)	99.25 ± 8.50; 185.88 (2–378)	1
Body mass index (normal values)	9	3	0.242
Nasal polyposis (presence)	1	2	0.242
Diabetes mellitus (presence)	2	—	0.524
Osteoporosis (presence)	1	2	0.242
Pancreatic insufficiency (presence)	10	3	0.095
Meconium ileus (presence)	3	—	0.505
*Pseudomonas aeruginosa *	6	4	0.600
Mucoid *P. aeruginosa *	4	4	0.282
*Achromobacter xylosoxidans *	2	—	0.524
*Burkholderia cepacia *	3	1	1
*Staphylococcus aureus *	9	3	0.242
Weight (kg)	41.50 ± 17.51; 36 (21–66)	48 ± 16.98; 50 (27–70)	0.513
Height (m)	1.74 ± 0.43; 2 (1-2)	1.50 ± 0.53; 1.50 (1-2)	0.594
Body mass index	18.54 ± 3.18; 19.95 (14.38–21.60)	18.25 ± 2.56; 18.48 (14.34–21.31)	0.768
SpO_2_	96.10 ± 1.45; 96 (94–98)	96 ± 2.31; 96 (94–98)	1
Bhalla	8 ± 4.74; 7 (0–17)	12.50 ± 3.53; 12.50 (10–15)	0.327
Kanga	19 ± 4.82; 19 (12–25)	30.50 ± 20.17; 23.50 (15–60)	0.260
Shwachman-Kulczycki	68.50 ± 13.34; 65 (50–90)	71.67 ± 10.40; 75 (60–80)	0.811
FVC	91.80 ± 20.02; 87.50 (69–131)	70.20 ± 20.17; 63 (57–106)	**0.028**
FEV_1_	85.10 ± 22.41; 75.50 (64–132)	59 ± 25.40; 60 (30–95)	0.055
FEV_1_/FVC	82.60 ± 18.92; 85.50 (37–100)	67.75 ± 17.58; 71 (46–83)	0.106
FEF_25–75_%	71.40 ± 30.85; 66 (33–118)	45.50 ± 24.78; 47.50 (17–70)	0.188

^*^The data are shown as *N* (percentage) for the categorical data; the statistical analysis consisted of the *χ* ^2^ test and Fisher's exact test; the mean ± standard deviation and median (minimum and maximum) values were used for the numerical data that was analyzed by the Mann-Whitney *U* test. *N*: number of patients; SpO_2_: blood oxygen saturation; FVC: forced vital capacity; FEV_1_: forced expiratory volume in the first second; FEF_25–75_%: forced expiratory flow between 25 and 75% of the FVC. G1: cystic fibrosis patients with two class I, II, or III CFTR mutations; G2: cystic fibrosis patients with at least one class IV, V, or V *CFTR* mutation.

**Table 3:** *CFTR* mutations found in the individuals under study. Gene and protein localization. Mutation classification and frequency from the present study are designated. Traditional and HGVS standard nomenclature^a^ for *CFTR* mutations are also indicated.

Traditional nomenclature	HGVS nomenclature^b^		Localization (*CFTR* gene)^c^	Consequence	Protein localization	Mutation classification	Predicted functional class
	cDNA name	Protein name					
F508del	c.1521_1523delCTT	p.Phe508del	Exon 10	Point deletion	NBD1	A	II
G542X	c.1624G>T	p.Gly542X	Exon 11	Nonsense	NBD1	A	I
P205S	c.613C>T	p.Pro205Ser	Exon 6a	Missense	TM3	A	IV
1717-1G>A	c.1585-1G>A	—	IVS11	Splicing	—	A	I
1812-1G>A	c.1680-1G>A	—	IVS12	Splicing	—	A	I
3272-26A>G	c.3140-26A>G	—	IVS17b	Splicing	—	A	V
V562I	c.1684G>A	p.Val562Ile	Exon 12	Missense	NBD1	B	—
D1152H	c.3454G>C	pAsp1152Hist	Exon 21	Missense	NBD2	A	IV
IVS8-5T	—	—	Intron 8	Splicing	—	A	V

A: CF-causing mutation; B: CFTR-RD mutation; C: mutation with no clinical consequence.

^a^Reference CFTR sequence accession number: NM_000492.3; nucleotide number 1 corresponds to the A of the ATG translation initiation codon; in the reference sequence, it is numbered as 133.

^b^According to the HVGS guidelines, this mutation should be named 1585–9412 bp A>G.

^c^Traditional nomenclature.

**Table 4:** Association of nasal potential difference between cystic fibrosis patients and healthy subjects.

NPD variables	Groups	*N*	Mean	Standard deviation	Standard error	Confidence interval		Minimum	Maximum	^#^ *P* value	*P* ^corrected^
						5%	95%				
Finger (mV)	A	10	−31.40	27.452	8.681	−51.04	−11.76	−57	37	*0.004 *	*0.020 *
	B	5	−39.40	7.956	3.558	−49.28	−29.52	−52	−31		
	C	21	−20.10	16.078	3.508	−27.41	−12.78	−41	36		
	Total	**36**	**−25.92**	**20.090**	**3.348**	**−32.71**	**−19.12**	**−57**	**37**		

PDMax (mV)	A	10	−2.40	18.075	5.716	−15.33	10.53	−42	21	0.111	0.555
	B	5	5.60	14.064	6.290	−11.86	23.06	−12	23		
	C	21	10.52	11.378	2.483	5.34	15.70	−14	28		
	Total	**36**	**6.25**	**14.594**	**2.432**	**1.31**	**11.19**	**−42**	**28**		

Δamiloride (mV)	A	10	12.30	18.379	5.812	−0.85	25.45	−20	42	0.611	1
	B	5	5.20	15.353	6.866	−13.86	24.26	−10	27		
	C	21	9.52	10.482	2.287	4.75	14.29	−5	45		
	Total	**36**	**9.69**	**13.469**	**2.245**	**5.14**	**14.25**	**−20**	**45**		

Δchloride-free + isoproterenol	A	10	0.750	7.270	2.299	−4.450	5.950	−12.0	12.5	*0.016 *	0.08
	B	5	−1.800	5.805	2.596	−9.008	5.408	−7.0	7.0		
	C	21	−7.881	8.235	1.7971	−11.630	−4.132	−26.5	6.0		
	Total	**36**	**−4.639**	**8.482**	**1.4137**	**−7.509**	**−1.769**	**−26.5**	**12.5**		

Wilschanski index	A	10	1.112	0.627	0.198	0.664	1.561	0.535	2.718	*0.005 *	*0.025 *
	B	5	0.610	0.274	0.123	0.269	0.950	0.223	0.883		
	C	21	0.435	0.362	0.081	0.267	0.603	0	1.051		
	Total	**36**	**0.648**	**0.526**	**0.088**	**0.469**	**0.826**	**0**	**2.718**		

*N* = number of patients; CF = cystic fibrosis; mV = millivolts; PDMax = maximum NPD; Wilschanski index = *e* ^Δchloride/Δamiloride^.

^#^Kruskal-Wallis statistical test.

The positive *P* value is in italic.

The *P* value was corrected using the Bonferroni test. Five analyses were performed on the same sample group.

## Abbreviations

CF: Cystic fibrosis
CFTR: Cystic fibrosis transmembrane regulator
DNA: Deoxyribonucleic acid
IRT: Immunoreactive trypsin
MLPA: Multiplex ligation-dependent probe amplification
MIM: Monogenic inheritance disease
NPD: Nasal potential difference.
